# Array based characterization of a terminal deletion involving chromosome subband 15q26.2: an emerging syndrome associated with growth retardation, cardiac defects and developmental delay

**DOI:** 10.1186/1471-2350-9-2

**Published:** 2008-01-14

**Authors:** Josef Davidsson, Anna Collin, Gudrun Björkhem, Maria Soller

**Affiliations:** 1 Department of Clinical Genetics, Lund University Hospital, Lund, Sweden; 2 Department of Pediatrics, Lund University Hospital, Lund, Sweden

## Abstract

**Background:**

Subtelomeric regions are gene rich and deletions in these chromosomal segments have been demonstrated to account for approximately 2.5% of patients displaying mental retardation with or without association of dysmorphic features. However, cases that report de novo terminal deletions on chromosome arm 15q are rare.

**Methods:**

In this study we present the first example of a detailed molecular genetic mapping of a de novo deletion in involving 15q26.2-qter, caused by the formation of a dicentric chromosome 15, using metaphase FISH and tiling resolution (32 k) genome-wide array-based comparative genomic hybridization (CGH).

**Results:**

After an initial characterization of the dicentric chromosome by metaphase FISH, array CGH analysis mapped the terminal deletion to encompass a 6.48 megabase (Mb) region, ranging from 93.86–100.34 Mb on chromosome 15.

**Conclusion:**

In conclusion, we present an additional case to the growing family of reported cases with 15q26-deletion, thoroughly characterized at the molecular cytogenetic level. In the deleted regions, four candidate genes responsible for the phenotype of the patient could be delineated: *IGFR1, MEF2A, CHSY1*, and *TM2D3*. Further characterization of additional patients harboring similar 15q-aberrations might hopefully in the future lead to the description of a clear cut clinically recognizable syndrome.

## Background

The subtelomeric regions of chromosomes are particularly gene rich [1] and deletions in these regions have been demonstrated to account for approximately 2.5% of patients displaying mental retardation with/without association of dysmorphic features [2]. The small interstitial deletion on the long arm of chromosome 15 causing Prader-Willi/Angelman (P-W/A) syndrome is well documented [3] whereas cases that report de novo terminal deletions on 15q are rare. Excluding cases displaying various ring structures that result in different sized deletions of chromosome 15 or unbalanced reciprocal translocations involving distal parts of 15q, only 12 cases have been reported harboring pure distal 15q26 deletions [4-14]. These cases display various degrees of clinical features including intrauterine growth retardation, complex cardiac abnormalities, developmental delay, clinodactyly, ear abnormalities, lung hypoplasia, microcephaly, diaphragmatic hernia, high arched palate, and hypoplastic kidneys. Reported survival of patients varied from 20 minutes to 12 years [12].

Previous mapping studies have by means of fluorescent in situ hybridization (FISH) demonstrated that terminal 15q-deletion frequently results in loss of one *IGF1R *allele. It has been postulated that a dosage effect of the *IGF1R *gene might be direct responsible for the intrauterine growth retardation seen in patients monosomic for 15q26 and for the postnatal overgrowth seen in patients trisomic for 15q25-qter [15]. However in vitro studies on fibroblast cells have been ambiguous. For instance Siebler et al. (1999) failed to provide conclusive evidence for a biological response to lowered *IGFR1 *expression due to distal deletion of 15q whereas Okubo et al. demonstrated accelerated and decreased growth in cells with three or one copy of the gene, respectively [5,8].

In this report we present a detailed genetic mapping including classical cytogenetics, metaphase FISH and tiling resolution (32 k) genome-wide array-based comparative genomic hybridization (array CGH) of a patient with a de novo deletion of subband 15q26.2, caused by the formation of a dicentric chromosome 15. Only one similar case with a karyotype harboring a bisatellite dicentric chromosome, resulting in a distal 15q deletion, has been reported previously in the literature [6].

## Case Report

The patient is the first child to healthy parents (maternal age 29 years) and was born at the 37^th ^week of pregnancy. APGAR score was 8-9-9. The birth-weight was 2.4 kg, borderline to small for gestational age. Single umbilical artery was not present. Growth and height centiles were ≤3 for the patient both at 1, 18 and 22 months of age. The child was first examined in the 33^rd ^gestational week, when fetal screening had indicated abnormal heart size. Fetal echocardiography showed a clearly enlarged right ventricle and a narrow left ventricle with a slightly small mitral valve. There was suspicion of a ventricular septal defect (VSD). The aortic arch could not be completely elucidated. A magnetic resonance tomography (MRT) was made of the fetus, but no further information was obtained. Neonatal echocardiography confirmed a large right ventricle, a somewhat underdeveloped left side, and gave a strong suspicion of coarctation of the aorta. There was also a small VSD. Treatment with prostaglandin was started and the child was put on ventilator. MRT showed a coarctation and an abnormal aortic arch with a very acute angle between the ascending and the descending aorta. The child was operated at the age of three days with resection of the coarctation and end-to-side anastomosis. The postoperative period was uneventful except for bouts of supraventricular tachycardia that were treated with adenosine and digitalis. X-ray showed relaxation of the right side of the diaphragm. Because of the patient's unusual facial features with low set ears, small chin, and haemangioma on the forehead, a chromosome analysis was performed on a blood sample drawn at day five.

**Figure 1:**
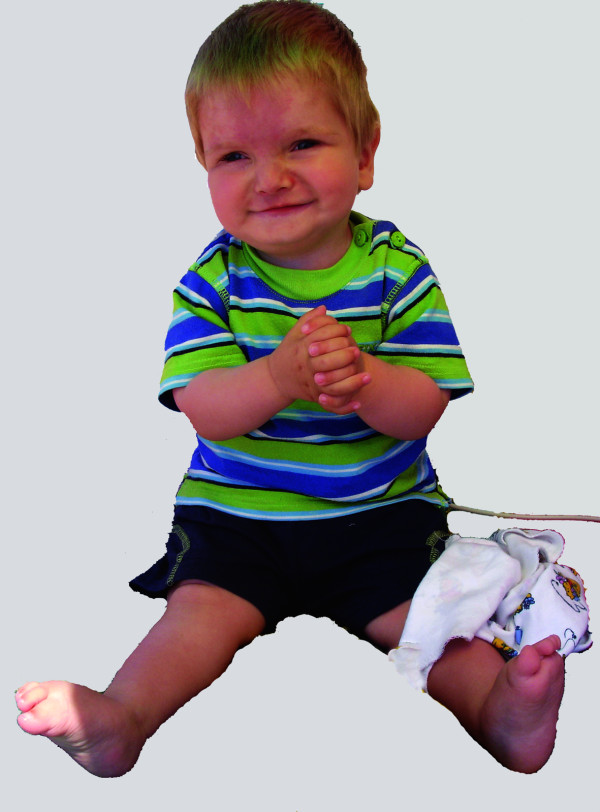
**Patient**. Phenotypic characteristics of the patient at 20 months.

At the age of 18 months the child is small with a weight of 7,7 kg and a length of 70 cm. The patient's physical and mental developmental milestones are severely delayed. He has been operated with a percutaneous gastrostomy and takes hardly any food by mouth, harboring problems with vomiting and gastrooesophageal reflux. At the lastest follow up the patients cardiac status was fairly good, indicating favorable result of the coarctation operation, but displayed signs of a moderate mitral stenosis and a remaining small VSD. He displays dysmorphic features like hypertelorism, rocker bottom feet, broad nasal bridge, narrow upper lip and flat philtrum (Figure 1).

## Methods

Blood samples from the patient and his parents were drawn after informed consent. Lymphocyte culture upon phytohemagglutinin stimulation and G-banding analysis was performed in standard clinical settings on the three samples. Both parents displayed normal karyotypes.

An initial analysis of the derivative chromosome detected in the patient was conducted with a whole chromosome paint (WCP) probe and a centromere probe specific for chromosome 15 (Vysis, Downers Grove, US) as well as with a probe specific for the nucleolus organizing regions (NOR) of the acrocentric chromosomes (Chrombios, Raubling, Germany). A probe specific for the P-W/A region (LSI D15S10/CEP15/LSI PML; Vysis) was also used. For a refined analysis of the derivative chromosome, a total of 12 bacterial artificial chromosome (BAC) probes targeting the 15q11.2-q14 region and the 15q25.1-q26.3 region, were selected from the UCSC Genome Browser [16] (Table 1). BAC DNA was extracted by alkaline lysis with SDS using standard protocols and labeled with Cy3-dUTP (GE Healthcare Bio-Sciences Corporation, Piscataway, NJ) or Fluorescein-12-dUTP (FITC) (Roche, Mannheim, Germany) using the Megaprime DNA Labelling System (GE Healthcare Bio-Sciences Corporation). Fixed metaphase spreads were aged over night at 60°C then pretreated with 20 mg/ml pepsin (Serva, Heidelberg, Germany), postfixed in 1% formaldehyde and finally dehydrated in an ethanol series (70%, 85%, 100%). Commercial probes were hybridized according to the manufacturers' recommendations. For each BAC probe, a total of 90 ng labeled DNA was hybridized. After application of the probe solution and sealing of the metaphase spread, the preparations were denaturated on a hot plate for 4 min at 74°C and then incubated over-night in a humid chamber at 37°C. Post-hybridization washes were done in 0.4× SSC/0.05% Tween-20 (Sigma-Aldrich, St Louis, US) followed by dehydration in an ethanol series. For detection of the nuclei and evaluation of the fluorescent signals, the spreads were counterstained with 0.5 mg/ml 4,6-diamidino-2-phenylindole (DAPI; Sigma-Aldrich) and mounted in a 2% 1,4-diazabicyclo-[2,2,2]-octane (DABCO) solution (Sigma-Aldrich). Conventional epifluorescence microscopy was carried out with a Zeiss Axioplan2 imaging microscope (Zeiss, Jena, Germany) linked to a CytoVision Ultra system (Applied Imaging, San José, US). A minimum of 11 metaphases (median 17) were analyzed for each probe mixture.

**Table 1:** Information on the probes used and results of hybridizations

	*Probe information*		*Results of FISH analyses*	
**Probe name**	**Accession no.**	**Locus**	**Normal 15**	**dic(15;15)**

NOR	-	p-arm*	1 signal	2 signals
CEP15	-	15p11.1-q11.1	1 signal	2 signals
RP11-509A17	AC026495	15q11.2	1 signal	1 signal
RP11-228M15	AC091565	15q11.2	1 signal	1 signal
RP11-1071C22	AQ813791, AQ791136	15q11.2 *SNRPN*	1 signal	1 signal
RP11-89E18	AQ282759, AQ282775	15q12	1 signal	1 signal
RP11-165M18	AQ382104, AQ382108	15q13.1	1 signal	1 signal
RP11-303I17	AZ301277, AZ301278	15q13.2-13.3	1 signal	1 signal
RP11-194H7	AQ412869	15q14	1 signal	1 signal
RP11-91M4	AQ282682, AQ282685	15q25.1	1 signal	1 signal
RP11-90B9	AZ517977, AQ283516	15q25.2	1 signal	1 signal
RP11-60P2	AC105120	15q25.3	1 signal	1 signal
RP11-378B5	AZ301319, AZ301320	15q26.1	1 signal	1 signal
RP11-79C10	AZ519147, AQ581275	15q26.2	1 signal	No signal
RP11-90E5	AQ281493, AQ281496	15q26.3	1 signal	No signal

*The NOR probe hybridizes to the p-arm of all acrocentric chromosomes (13, 14, 15, 21, 22)

DNA was extracted from the peripheral blood cells obtained at the time of diagnosis. Male genomic DNA (Promega, Madison, WI) was used as reference in the hybridization. The 32 k slides used, containing 32 433 tiling BAC clones covering at least 98% of the human genome, were produced at the SWEGENE DNA microarray resource center at Lund University, Sweden. Labeling of DNA, slide preparation, and hybridization were performed as described [17] with minor modifications. Analysis of the microarray images were performed with the GenePix Pro 4.0 software (Axon Instruments, Foster City, CA). For each spot, the median pixel intensity minus the median local background for both dyes was used to obtain the ratio of test gene copy number to reference gene copy number. Data normalization was performed for each array subgrid using lowess curve fitting with a smoothing factor of 0.33 [18]. The sex chromosomes were excluded when calculating the correction factor in the normalization of the data set. All normalizations and analyses were performed in the Bioarray Software Environment database (BASE) [19]. To identify imbalances, the MATLAB toolbox CGH plotter and the TM4 software suite [20] was applied, using a moving mean average over three clones and log2 limits of ≥0.2. Classification as gain or loss was based on identification as such by the CGH plotter and also by visual inspection of the log2 ratios. Ratios +/- ≥0.5 in three adjacent clones were classified as abnormal, with ratios between 0.5 and 1.0 interpreted as duplications/hemizygous deletions and ≥1.0 classified as amplifications/homozygous deletions.

Written informed consent was obtained for publication of this study.

## Results

### Cytogenetics

**Figure 2:**
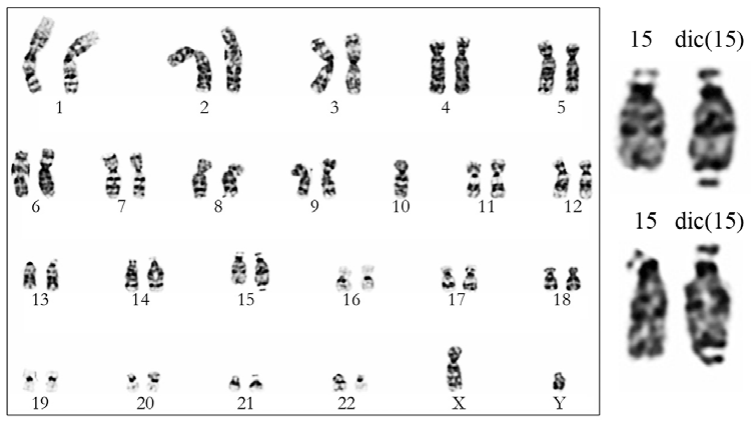
**Cytogenetics**. Representative karyogram of the patient displaying the detected dic(15) and normal 15 from two different karyotypes.

Cytogenetic studies from the peripheral blood of the two-months-old patient displayed a male karyotype with a dicentric chromosome 15: 46,XY,+15,dic(15;15)(q11;q26.1) (Figure 2). Maternal and paternal karyotypes were normal. The characterization of the derivative chromosome was expanded by FISH in order to elucidate the cytogenetic findings. The subsequent array CGH analysis was performed at a later stage after informed consent from the patients' parents.

### FISH

**Figure 3:**
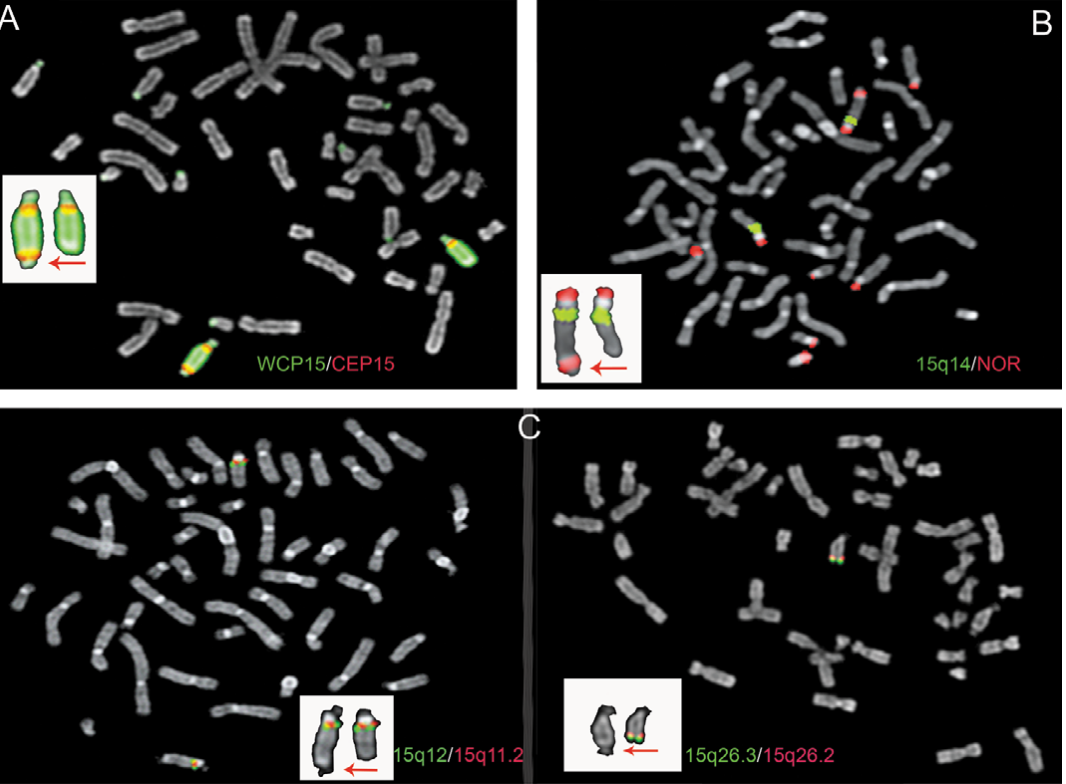
**Illustrative metaphase FISH images**. The derivative chromosome is indicated with an arrow. **A. **Hybridization with a chromosome 15 specific WCP probe and a centromere 15 (CEP15) specific probe indicating that the derivative chromosome was entirely composed of chromosome 15-material and that it was dicentric. WCP15 cross-hybridizes with the p-arms of all acrocentric chromosomes (seen as green spots). **B. **Hybridization with a probe specific for the nucleolus organizing regions (NOR) of the acrocentric chromosomes and a control probe (15q14) showing the presence of p-arm material in duplicate on the derivative chromosome. **C. **Hybridization with BAC probes specific for the 15q11.2-q13.2 and 15q25.1-q26.1 regions showed that the corresponding target area was present in one copy on the derivative chromosome (left). Hybridization with BAC probes specific for the 15q26.2-15q26.3 region showed that the corresponding regions were lacking on the derivative chromosome (right).

Hybridization with chromosome 15 specific WCP- and centromere probes indicated that the derivative chromosome was composed entirely of material from chromosome 15 and that it contained two centromeric regions, one at each end of the structure (Figure 3a). Hybridization with a NOR-specific probe showed a bright signal at each end of the structure implying the presence of p-arm material in duplicate on the derivative chromosome. The control probe RP11-194H7, hybridizing to15q14, was present only in one copy on the derivative chromosome (Figure 3b). Hybridization with the probe specific for the P-W/A locus was not conclusive (data not shown) but hybridization with the BAC probe RP11-1071C22 showed that *SNRPN *gene was not deleted indicating that P-W/A was not a putative diagnosis. Hybridization with additional 12 BAC probes revealed a normal hybridization pattern for the probes specific for the 15q11.2-q13.2 and 15q25.1-q26.1 regions, implicating that the corresponding chromosomal material was present only in one copy on the derivative chromosome. Hybridization with the BAC probes RP11-79C10 and RP11-90E5, specific for regions within 15q26.2 and 15q26.3, respectively, showed signals only on the normal chromosome 15 homologue implicating a lack of these regions on the derivative chromosome (Figure 3c). The results of the FISH analysis are summarized in Table 1.

### Array CGH

**Figure 4:**
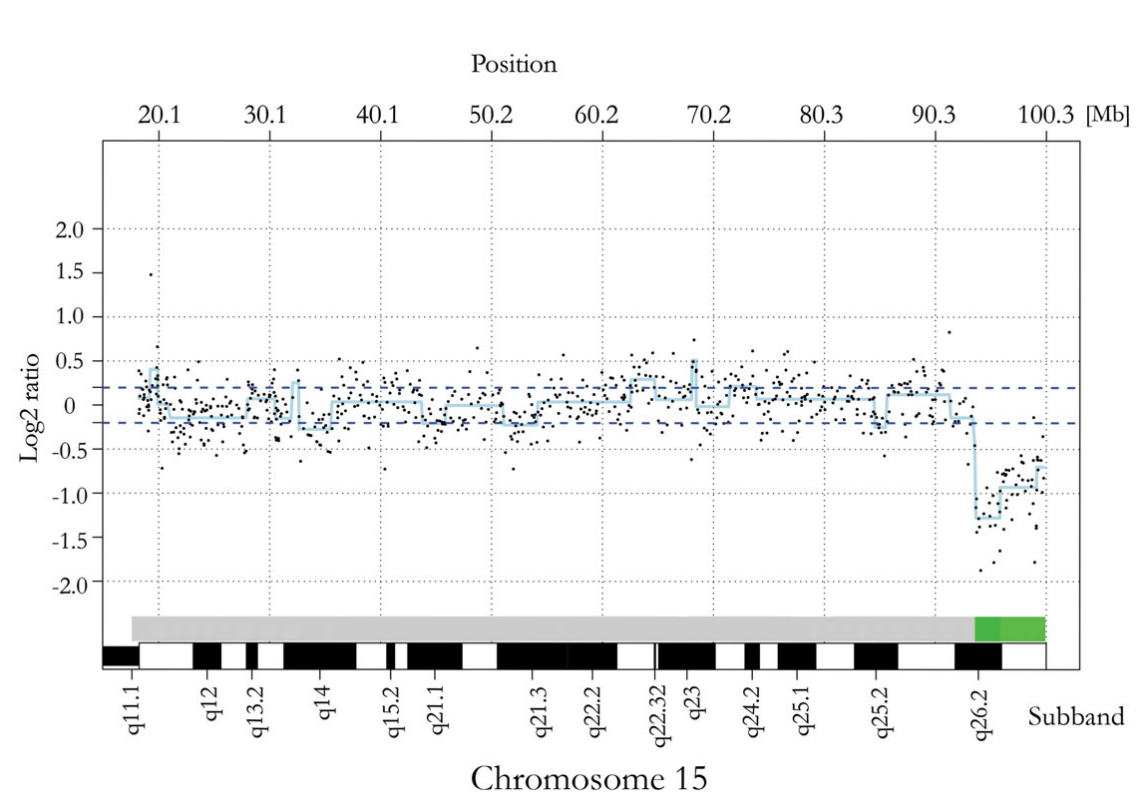
**Array CGH**. Log2 ratio plot for chromosome 15 displaying the deleted segment (green bar).

The array CGH analysis confirmed the distal deletion of 15q resulting from the dic(15) that was initially detected by the G-banding analysis. In concordance with the subsequent FISH findings the deletion breakpoint was revised to 15q26.2 instead of 15q26.1 (Figure 4), the breakpoint most frequently reported in the literature for similar cases and also diagnosed by our clinical cytogeneticists in the initial G-banding analysis (Figure 2). The array CGH analysis mapped the terminal deletion to encompass a 6.48 megabase (Mb) region, ranging from 93.86–100.34 Mb on chromosome 15. Known genes in the region are summarized in Table 2. No additional aberrations were detected.

**Table 2:** USCS known genes in 93.86–100.30 Mb on chr 15 based on RefSeq, Uniprot annotation, and Genbank mRNA

**Gene**	**Uniprot accession number**	**Basepair position on chr 15**	**Size in basepairs**
*IGF1R*	P08069	97010288–97319034	308746
*TTC23*	Q5W5X9	97494052–97607246	113194
*MEF2A*	Q59GX4	97956185–98071524	115354
*ADAMTS17*	Q8TE56	98331993–98699650	367657
*LINS1*	Q9NVQ3	98926957–98959902	32945
*ASB7*	Q6GSJ6	98960358–99007174	46816
*ALDH1A3*	Q96GT2	99237584–99274352	36768
*LRRK1*	Q6NYC0	99276983–99427840	150857
*CHSY1*	Q86X52	99533455–99609649	76194
*SELS*	Q6GYA4	99628737–99635223	6486
*SNRPA1*	P09661	99639238–99652983	13745
*TM2D3*	Q9BRN9	99999572–100010117	10545
*TARSL2*	Q6B0A1	100011478–100082168	70690
*OR4F4*	Q96R69	100279868–100280785	917

## Discussion

Constitutional deletions involving chromosome band 15q26 seem to be rather rare unbalanced aberrations which seldom are diagnosed and characterized at the cytogenetic level. However with subtelomeric FISH becoming standard clinical practice and array CGH being more commonly implicated as a powerful tool to characterize cases with submicroscopic aberrations it is likely to assume that patients harboring distal 15q-deletions might be more frequently discovered in the future.

In this study we used array CGH to delineate the breakpoint to position 93.86 Mb on chromosome 15. This was consistent with the preceding FISH analysis that localized the most proximally deleted BAC as clone RP11-79C10, positioned within subband 15q26.2, and encompassing the area detected by array CGH [21]. This approach of using tiling BACs to cover the whole genome in a single hybridization gives a resolution standard of 150–350 kb, applying the exclusion criteria that three contiguous clones must display abnormal log2 ratios to be considered to signal an aberration. This study, characterizing in detail a constitutional 15q26-deletion, is one of the first ones using molecular cytogenetic characterization with submegabase resolution on a syndrome related to this chromosomal region. Klaassens et al. (2005) used a similar approach relying on array CGH and FISH, but focused on cases presenting with distal 15q breakpoints resulting from ring chromosome formation or unbalanced translocations to determine a critical region associated with congenital diaphragmatic hernia (CDH). The platform used was a 1-Mb Human BAC array, giving resolution standards tenfold smaller than 32 k arrays [11]. However, a later study used array techniques with submegabase resolution on a similar patient [22]. In addition Slavotinek et al. (2005) have previously identified microdeletions at 15q26.2 to be associated with Fryns syndrome, a CDH-related autosomal recessive syndrome with relatively homogenous phenotype, using 2.4 k array [14].

The origin of the dicentric chromosome in the present case remains unknown since both parents displayed normal karyotypes and directed microsatellite analysis was not performed. However, it has been demonstrated that a de novo 15q-deletion can be of both paternal and maternal origin [4,6]. Studies on terminal chromosomal deletions have suggested that de novo telomere addition, by telomerase activity or recombinant mechanisms, work to stabilize the affected chromosome [23]. It could be speculated that the latter event might explain the formation of the dic(15) in the present case, but involving centromeres rather than telomeres. It is noteworthy that no duplication of near-centromeric q-arm material could be detected on the log2 ratio plot of chromosome 15 (Figure 4). The preceding FISH analysis had confirmed that the duplication included the centromeric region, as well as p-arm material, both being located in regions not covered by the array.

The vast majority of patients with deletions of distal 15q have displayed pre- and postnatal growth retardation, cardiac defects, developmental delay, ear abnormalities, and clinodactyly. A variety of other different dysmorphic features have however been reported in association with 15q26 deletions such as café-au-lait spots, cholestatic liver disease, diaphragmatic hernia, eye and face abnormalities, equinovarus, high arched palate, hypertelorism, hypoplastic kidneys, hypotonia, lung hypoplasia, microcephaly, micrognathia, and spinal cord abnormality. Although high resolution cytogenetic mapping for all reported cases are lacking this wide range of phenotypic manifestations probably reflect size heterogeneity of distal 15q-deletions. More precise data on 15q-breakpoints might enable an elucidation of different candidate regions for different phenotypic manifestations. For instance, patients suffering from CDH in association with 15q26-deletion have been extensively studied and a critical region associated with CDH was assessed to a region ranging from distal 15q26.1 to proximal 15q26.2 [11]. The 15q-breakpoint in the present case mapped outside of the suggested critical region and in accordance did not suffer from CDH. Deletions of 15q26 have also been reported in association with Russel-Silver syndrome, which share many of the clinical features displayed in patients with deletions of distal 15q and it is possible that these cases should be categorized as an subgroup of the latter syndrome [24,25].

Considering all the reported cases with deletion of 15q26, the salient finding must be that the majority report loss of *IGFR1*, a gene that contributes to a wide range of developmental processes [26]. The relation between *IGFR1 *and pre- and postnatal growth in association with distal 15q-deletions has been studied without conclusive results [5,8]. In relation to the developmental delay commonly seen in patients with distal 15q-deletions, the IGF1 receptor gene plays an important function in the development of the central nervous system, which could partly explain this feature [27]. Studies on the effect of *IGFR1 *on the cardiovascular system indicate that it is an important player in heart development [24]. Monozygosity of this gene could therefore partly contribute to the complex cardiac defects so often seen in patients with this deletion. Obviously, all suggested clinical features postulated to relate to loss of one copy of the *IGFR *gene remains to be elucidated, preferentially by further gene expression studies on diagnostic material.

*MEF2A *have been suggested as a candidate gene for the cardiac defects [11] included in the phenotype of 15q26-deletions since the gene encode for DNA-binding regulatory protein that enhances the differentiation of mesodermal precursor cells to myoblast and therefore is key player in cardiac myocyte development [28]. The MEF2-family is an ancient mediator of signal-dependant transcription and diverse developmental programs and *MEF2A *was quite recently demonstrated to be involved in morphogenesis of postsynaptic neurons [29] which implicate that it could also contribute to the developmental delay seen in the patient.

Another gene in the deleted segment that could be directly implicated due to its protein function is *CHSY1 *that encodes chondroitin sulfate [30], a glycosaminoglycan expressed on the surface of most cells and in extracellular matrices. Glycosaminoglycan chains are covalently linked to a wide range of core protein families and regulate many biologic processes, including cell proliferation and recognition, extracellular matrix deposition, and morphogenesis. Furthermore, *TM2D3*, located in the deleted region, synthesizes a protein that have a structural module related to that of the seven transmembrane domain G protein-coupled receptor superfamily and may have regulatory roles in cell death or proliferation signal cascades [31].

## Conclusion

We present a case with a dic(15;15)(q11;q26.1) which was thoroughly characterized with locus specific FISH and tiling resolution array CGH. The dicentric chromosome harbor a terminal deletion involving 15q26.2-qter a region that encompass 14 genes, among which four candidate genes could be delineated. *MEF2A *has previously been suggested as a candidate gene for the cardiac defects seen in investigated patients. In addition *CHSY1 *is involved in cell proliferation and morphogenesis and *TM2D3 *may have regulatory roles in cell death or proliferation signal cascades. Previous studies have demonstrated that terminal 15q-deletions frequently results in loss of one allele of the *IGF1R *gene, a finding also observed in our patient. *IGF1R *contributes to a wide range of developmental processes such as development of the central nervous and cardiovascular systems, and monozygosity for this gene could contribute to the developmental delay and complex cardiac defects seen in our patient. However, all suggested clinical features postulated to relate to *IGF1R *deletions remains to be elucidated, preferentially by gene expression studies on patients harboring similar 15q-aberrations. Hopefully, this may the future lead to the description of a clear cut clinically recognizable syndrome.

## Abbreviations

array CGH, array-based comparative genomic hybridization; BAC, bacterial artificial chromosome; CDH, congenital diaphragmatic hernia; FISH, Fluorescent In Situ Hybridization; NOR, nucleolus organizing regions; WCP, whole chromosome paint

## Competing interests

The author(s) declare that they have no competing interests.

## Authors' contributions

JD performed the array CGH, wrote and finalized the manuscript.

AC performed the FISH analyses and drafted the manuscript.

GB treated the patient and wrote the clinical case report

MS participated in genetic counseling, designed the study and drafted the clinical case report.

All authors have read and approved the final version of the manuscript.

## Pre-publication history

The pre-publication history for this paper can be accessed here:



## References

[B1] Saccone S, De Sario A, Della Valle G, Bernardi G (1992). The highest gene concentrations in the human genome are in telomeric bands of metaphase chromosomes. Proc Natl Acad Sci USA.

[B2] Ravnan JB, Tepperberg JH, Papenhausen P, Lamb AN, Hedrick J, Eash D, Ledbetter DH, Martin CL (2005). Subtelomere FISH analysis of 11688 cases: an evaluation of the frequency and pattern of subtelomere rearrangements in individuals with developmental disablities. J Med Genet.

[B3] Schinzel A (2001). Catalogue Of Unbalanced Chromosome Aberrations In Man 2'nd Revised And Expanded Edition.

[B4] Roback EW, Barakat AJ, Dev VG, Mbikay M, Chretien M, Butler MG (1991). An infant with deletion of the distal long arm of chromosome 15 (q26.1----qter) and loss of insulin-like growth factor 1 receptor gene. Am J Med Genet.

[B5] Siebler T, Lopaczynski W, Terry CL, Casella SJ, Munson P, De Leon DD, Phang L, Blakemore KJ, McEvoy RC, Kelley RI (1995). Insulin-like growth factor I receptor expression and function in fibroblasts from two patients with deletion of the distal long arm of chromosome 15. J Clin Endocrinol Metab.

[B6] Whiteford ML, Baird C, Kinmond S, Donaldson B, Davidson HR (2000). A child with bisatellited, dicentric chromosome 15 arising from a maternal paracentric inversion of chromosome 15q. J Med Genet.

[B7] Tönnies H, Schulze I, Hennies H-C, Neumann LM, Keitzer R, Neitzel H (2001). De novo terminal deletion of chromosome 15q26.1 characterised by comparative genomic hybridisation and FISH with locus specific probes. J Med Genet.

[B8] Okubo Y, Siddle K, Firth H, O'Rahilly S, Wilson LC, Willatt L, Fukushima T, Takahashi S-I, Petry CJ, Saukkonen T, Stanhope R, Dunger DB (2003). Cell proliferation activities on skin fibroblasts from a short child with absence of one copy of the type 1 insulin-like growth factor receptor (IGF1R) gene and a tall child with three copies of the IGF1R gene. J Clin Endocrinol Metab.

[B9] Hengstschläger M, Mittermayer C, Repa C, Drahonsky R, Deutinger J, Bernaschek G (2003). Association of deletion of the chromosomal region 15q24-ter and diaphragmatic hernia: a new case and discussion of the literature. Fetal Diagn Ther.

[B10] Biggio JR, Descartes MD, Carroll AJ, Holt RL (2004). Congenital diaphragmatic hernia: is 15q26.1-26.2 a candidate locus?. Am J Med Genet A.

[B11] Slavotinek A, Lee SS, Davis R, Shrit A, Leppig KA, Rhim J, Jasnosz K, Albertson D, Pinkel D (2005). Fryns syndrome phenotype caused by chromosome microdeletions at 15q26.2 and 8p23.1. J Med Genet.

[B12] Bhakta KY, Marlin SJ, Shen JJ, Fernandes CJ (2005). Terminal deletion of chromosome 15q26.1: case report and brief literature review. J Perinatol.

[B13] Klaassens M, van Dooren M, Eussen HJ, Douben H, den Dekker AT, Lee C, Donahoe PK, Galjaard RJ, Goemaere N, de Krijger RR, Wouters C, Wauters J, Oostra BA, Tibboel D, de Klein A (2005). Congenital diaphragmatic hernia and chromosome 15q26: determination of a candidate region by use of fluorescent in situ hybridization and array-based comparative genomic hybridization. Am J Hum Genet.

[B14] Pinson L, Perrin A, Plouzennec C, Parent P, Metz C, Collet MJ, Le Bris MJ, Douet-Guilbert N, Morel F, De Braekeleer M (2005). Detection of an unexpected subtelomeric 15q26.2--> qter deletion in a little girl: clinical and cytogenetic studies. Am J Med Genet A.

[B15] Nagai T, Shimokawa O, Harada N, Sakazume S, Ohashi H, Matsumoto N, Obata K, Yoshino A, Murakami N, Murai T, Sakuta R, Niikawa N (2002). Postnatal overgrowth by 15q-trisomy and intrauterine growth retardation by 15q-monosomy due to familial translocation t(13;15): dosage effect of IGF1R?. Am J Med Genet.

[B16] UCSC Genome Browser. http://genome.ucsc.edu/.

[B17] Jönsson G, Staaf J, Olsson E, Heidenblad M, Vallon-Christersson J, Osoegawa K, de Jong P, Oredsson S, Ringner M, Höglund M, Borg Å (2007). High-resolution genomic profiles of breast cancer cell lines assessed by tiling BAC array comparative genomic hybridization. Genes Chromosomes Cancer.

[B18] Yang YH, Dudoit S, Luu P, Lin DM, Peng V, Ngai J, Speed TP (2002). Normalization for cDNA microarray data: a robust composite method addressing single and multiple slide systematic variation. Nucleic Acids Res.

[B19] Saal LH, Troein C, Vallon-Christersson J, Gruvberger S, Borg Å, Peterson C (2002). BioArray Software Environment (BASE): a platform for comprehensive management and analysis of microarray data. Genome Biol.

[B20] Saeed AI, Sharov V, White J, Li J, Liang W, Bhagabati N, Braisted J, Klapa M, Currier T, Thiagarajan M, Sturn A, Snuffin M, Rezantsev A, Popov D, Ryltsov A, Kostukovich E, Borisovsky I, Liu Z, Vinsavich A, Trush V, Quackenbush J (2003). TM4: a free, open-source system for microarray data management and analysis. Biotechniques.

[B21] The BAC Resource Consortium (2001). Integration of cytogenetic landmarks into the draft sequence of the human genome. Nature.

[B22] Scott DA, Klaassens M, Holder AM, Lally KP, Fernandes CJ, Galjaard RJ, Tibboel D, de Klein A, Lee B (2007). Genome-wide oligonucleotide-based array comparative genomic hybridization analysis of non-isolated congenital diaphragmatic hernia. Hum Mol Genet.

[B23] Varley H, Di S, Scherer SW, Royle NJ (2000). Characterization of terminal deletions at 7q32 and 22q13.3 healed by de novo telomere addition. Am J Hum Genet.

[B24] Wilson GN, Sauder SE, Bush M, Beitins IZ (1985). Phenotypic delineation of ring chromosome 15 and Russell-Silver syndromes. J Med Genet.

[B25] Tamura T, Tohma T, Ohta T, Soejima H, Harada N, Abe K, Niikawa N (1993). Ring chromosome 15 involving deletion of the insulin-like growth factor 1 receptor gene in a patient with features of Silver-Russell syndrome. Clin Dysmorphol.

[B26] Delafontaine P, Song YH, Li Y (2004). Expression, regulation, and function of IGF-1, IGF-1R, and IGF-1 binding proteins in blood vessels. Arterioscler Thromb Vasc Biol.

[B27] Woods KA, Camacho-Hubner C, Savage MO, Clark AJ (1996). Intrauterine growth retardation and postnatal growth failure associated with deletion of the insulin-like growth factor I gene. N Engl J Med.

[B28] Potthoff MJ, Olson EN (2007). MEF2: a central regulator of diverse developmental programs. Development.

[B29] Shalizi A, Gaudillière B, Yuan Z, Stegmüller J, Shirogane T, Ge Q, Tan Y, Schulman B, Harper JW, Bonni A (2006). A calcium-regulated MEF2 sumoylation switch controls postsynaptic differentiation. Science.

[B30] Kitagawa H, Izumikawa T, Uyama T, Sugahara K (2003). Molecular cloning of a chondroitin polymerizing factor that cooperates with chondroitin synthase for chondroitin polymerization. J Biol Chem.

[B31] Mahr S, Burmester GR, Hilke D, Göbel U, Grützkau A, Häupl T, Hauschild M, Koczan D, Krenn V, Neidel J, Perka C, Radbruch A, Thiesen HJ, Müller B (2006). Cis- and trans-acting gene regulation is associated with osteoarthritis. Am J Hum Genet.

